# Supplementary material for: Drugs Commonly Applied to Kidney Patients May Compromise Renal Tubular Uremic Toxins Excretion
Source: Toxins (Basel). 2020 Jun 12;12(6):391. doi: 10.3390/toxins12060391 (PMC7354492; doi:10.3390/toxins12060391)
Supplement: Supplementary file 1 [file toxins-12-00391-s001.pdf]

Supplementary Materials: Drugs Commonly Applied to Kidney Patients May Compromise Renal Tubular Uremic Toxins Excretion

Silvia M. Mihaila, João P. Ferreira Faria, Maurice F.J. Stefens, Dimitrios Stamatialis, Marianne C. Verhaar, Karin G.F. Gerritsen and Rosalinde Masereeuw

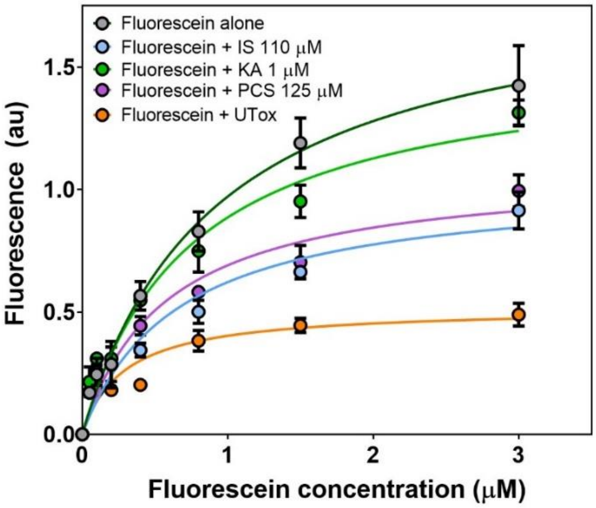

Figure S1. Concentration-dependent OAT1-mediated uptake of fluorescein (0–3μM) after 10 min incubation in ciPTEC-OAT1 in the presence of selected uremic toxins**.** The curves (*n* = 3) were fitted according to a Michaelis-Menten model.

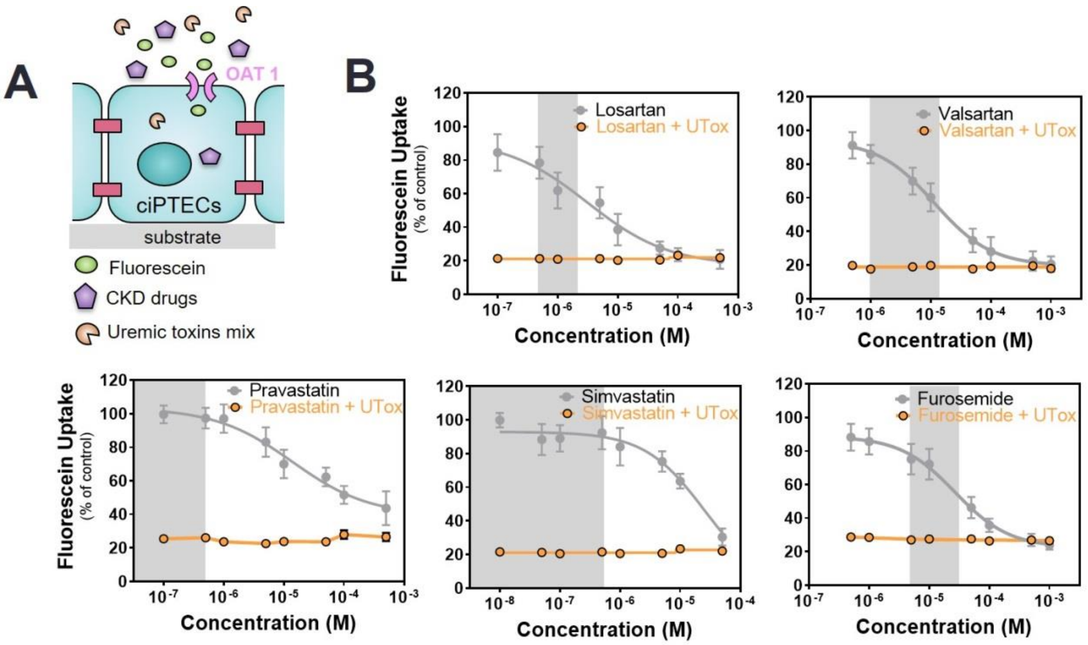

Figure S2. Interaction between drugs and uremic toxins mix (UTox) to inhibit OAT1-mediated fluorescein uptake. (**A**) Schematic representation of the co-incubation of fluorescein with variable concentrations of drugs and UTox. (**B**) The drug alone (grey line) shows a concentration-dependent inhibition effect. The

incubation with UTox alone results in a strong inhibitory effect, which is maintained during co-incubation with selected drugs (orange line). Grey regions indicate the therapeutic window of the respective drug.

Table S1. Concentrations of PBUTs used in the present study (individual and in UTox). Concentrations are adapted from EUToX Uremic Solutes Database [42] and Jansen et al. [37].

**Compound**		**Concentration (µM)**	
	Individual		
Indoxyl sulfate		110	
Kynurenic acid		1	
p-Cresylsulfate		125	
Uremic toxin mix (UTox)	Uremic concentration (µM)		IC_{50} (µM) [37]
Indoxyl sulfate	173.5 ± 121.9	100	25 ± 4
Indoxyl-β-D-glucoronide	9.4 ± 9.4	10	492 ± 68
Indole-3-acetic acid	11.4 ± 2.3	10	19 ± 2
Kynurenic acid	0.8 ± 0.4	1	6 ± 1
L-Kynurenine	3.3 ± 0.9	5	65 ± 8
Hippuric acid	608.4 ± 362.8	300	5 ± 1
p-Cresylglucuronide	30.1 ± 6.7	40	2650 ± 922
p-Cresylsulfate	122.2 ± 90.3	125	79 ± 14

Table S2. Michaelis-Menten parameters for concentration-dependent OAT1-mediated fluorescein (FL) uptake in ciPTEC-OAT1 in the presence of selected uremic toxins.

**Compound**	**Compound Concentration (µM)**	**K_m (µM)**	**V_{max} (au)**	**R square**
FL alone	0–3	0.93 ± 0.09	1.87 ± 0.07	0.971
FL + IS	110	0.66 ± 0.10	1.03 ± 0.06	0.907
FL + KA	1	0.75 ± 0.08	1.55 ± 0.67	0.954
FL + pCS	125	0.57 ± 0.07	1.09 ± 0.49	0.938
FL + UTox	mix	0.29 ± 0.03	0.52 ± 0.03	0.852

Abbreviations: FL = fluorescein; IS = indoxyl sulfate; KA = kynurenic acid; PCS = p-cresylsulfate, UTox = uremic toxin mix.

Table S3. Panel of selected drugs tested for their inhibitory effect over the fluorescein OAT1-mediated uptake in ciPTEC-OAT1: characteristics and range of concentrations used within the study.

Drug	Molecular Weight (g/mol)	Catalog Number (Sigma)	Stock Concentration	Solvent	Range of Concentrations
		ACEIs			
Captopril	217	C4042	100 mM	MilliQ	500 nM– mM
Enalaprilate	384	E9658	10 mM	MilliQ	500 nM–1 mM
Lisinopril	441	L0702000	50 mM	MilliQ	100 nM–500 μM
		ARBs			
Valsartan	435	SML0142	10 mM	DMSO	50 nM–1 mM
Losartan	422	Y0001062	50 mM	DMSO	100 nM–500 μM
		Statins			
Simvastatin	418	S6196	5 mM	DMSO	10 nM–50 μM
Pravastatin	446	P4498	50 mM	MilliQ	100 nM–500 μM
		Diuretics			
Furosemide	330	F4381	100 mM	DMSO	500 nM–1 mM
		H2RA			
Cimetidine	252	C4522	150 mM	MilliQ	500 nM–1 mM

Abbreviations: ACEIs = angiotensin-converting enzyme inhibitors; ARBs = angiotensin receptor blockers; H2RA = histamine H_2 receptor antagonists.

Table S4. Overview of the percentage (%) reduction of fluorescein uptake in the presence of UTox and drugs (at the lowest concentration).

Toxin	Percentage (%) Fluorescein Uptake at the Lowest Drug Concentration					
	No Drug	ARBs		Statins		Diuretics
	Reference	Losartan	Valsartan	Pravastatin	Simvastatin	Furosemide
**UTox**	34.4 ± 8.3	23.5 ± 3.5	24.7 ± 4.5	27.8 ± 2.6	25.7 ± 2.9	28.7 ± 3.2

Abbreviations: UTox = uremic toxin mix; ARBs = angiotensin receptor blockers.

Table S5. Physicochemical determinants of human renal clearance of tested drugs.

Drug	Total CL (mL/min/kg) [78]	Renal CL (mL/min/kg) [78]	Renal Clearance (% of Total CL)	Protein Binding (%) (www.drugbank.ca)	$T_{1/2}$ (h) [44]
		ACEIs			
Captopril	12	12	100	25–30	1–2
Enalaprilate	1.6	1.6	100	50	8–11
Lisinopril	1.2	1.2	100	negligible	12
		ARBs			
Losartan	8.2	0.9	11	99	1.5–2
Valsartan	0.49	0.14	29	94–97	6–9
		Diuretics			
Furosemide	2.4	1.7	71	95–99	1–3
		Statins			
Pravastatin	14	6.3	45	60	1–2.5 [45]
Simvastatin	–	–	13	95	2
		H2RA			
Cimetidine	8.1	7.9	97	20	1.5–4

Abbreviations: CL = clearance; ARBs = angiotensin receptor blockers; H2RA = histamine H_2 receptor antagonists, $T_{1/2}$= pharmacologic half life

Table S6. Overview of the lowest drug concentrations at which a statistically significant decrease ($p<0.05$) of the percentage (%) of fluorescein uptake was observed.

**PBUTs**	**Drug Concentration (μM)**					
	**No Drug**	**ARBs**		**Statins**		**Diuretics**
	**% FL Uptake Reference Values**	**Losartan**	**Valsartan**	**Pravastatin**	**Simvastatin**	**Furosemide**
IS	44.0 ± 5.4	1 *	5 *	5	5	50
KA	88.2 ± 8.5	5	10 *	5	10	5 *
PCS	46.7 ± 4.5	1 *	1 *	1 *	1 *	5 *

* indicates concentrations that fall in the therapeutic concentration range. Abbreviations: FL = fluorescein; PBUTs = protein-bound uremic toxins; IS = indoxyl sulfate; KA = kynurenic acid; PCS = p-cresylsulfate; ARBs = angiotensin receptor blockers.
